# Unlocking insights from actigraphy: examining feature selection and activation detection approaches for enhanced data interpretation

**DOI:** 10.1192/j.eurpsy.2024.1594

**Published:** 2024-08-27

**Authors:** S. László, Á. Nagy, J. Dombi, M. P. Fülep, E. Rudics, E. Hompoth, Z. Szabó, A. Dér, A. Buzás, Z. J. Viharos, A. T. Hoang, V. Bilicki, I. Szendi

**Affiliations:** ^1^Doctoral School of Interdisciplinary Medicine; ^2^Department of Software Engineering; ^3^Department of Computer Algorithms and Artificial Intelligence, University of Szeged; ^4^Institute of Biophysics, ELKH Biological Research Centre, Szeged; ^5^Center of Excellence in Production Informatics and Control, Institute for Computer Science and Control, Budapest; ^6^Department of Psychiatry, Kiskunhalas Semmelweis Hospital, Kiskunhalas; ^7^Department of Personality, Clinical and Health Psychology, University of Szeged, Szeged, Hungary

## Abstract

**Introduction:**

Alterations in motor activity are an extremely important characteristic and one of the leading symptoms of major functional psychiatric disorders. These pattern disturbances can be observed in schizophrenia. Actigraphy is a non-invasive method that can be used to monitor these changes, and recent studies emphasize its significance in the early identification of disorders like schizophrenia.

**Objectives:**

This study uniquely focuses on distinguishing latent liabilities for schizotypy from manifested schizophrenia using specific actigraphy features.

**Methods:**

Actigraphy data were collected using specialized devices from the University of Szeged and Haukeland University Hospital datasets (Berle et al., 2010). At Haukeland University Hospital patients with chronic schizophrenia (N=23) (so-called: manifested group) were collected, separately, at the University of Szeged, healthy university students were recruited and screened for latent tendencies towards shizotypic pathological development. In the latter study, two main groups were formed based on their scores: a positive schizotypy factor group (so-called: latent group) (N=22) and a control group (N=25), with actigraphy data.

Utilizing the pyActigraphy library (Hammad et al., 2021) and wavelet analysis, features such as activity mean, interdaily stability and sleep movement characteristics were derived. Feature selection employed machine learning algorithms, notably Logistic Regression, Random Forest, ANN, and AHFS aided by Shapley values and Click Forming Feature Selection for insight into the most influential features.

**Results:**

The three models exhibited similar performance with a 60% accuracy threshold. In the latent group, sleep-related movements have a substantial impact, while in the manifested group, in addition to sleep characteristics, features like RA, IV, ADAT, M10, the mean activity level (all of which decreased), and the ratio of zero values also play a significant role. In the latent group, features related to the length of small amplitude movements were dominant, particularly the increased values, along with a decrease in the density of large movements.

**Conclusions:**

Our study indicates that in the latent phase of schizophrenia, actigraphy features related to sleep are most significant, but as the disease progresses, both sleep and daytime activity patterns are crucial. Sleep disturbances may signal early susceptibility, with nighttime movements offering clearer insights. These variations might be influenced by medication effects in the manifested group, reflecting the broader challenges in schizophrenia research where the drug-free study of patients remains elusive. Further studies should explore these features in the Clinical High Risk and prodromal groups to refine our understanding of the development of the disorder.

**Disclosure of Interest:**

None Declared

